# Examining Associations Between Legal Frameworks for Medical Planning and Intensity of End-of-Life Care

**DOI:** 10.1093/geroni/igaa057.217

**Published:** 2020-12-16

**Authors:** Melissa Berkowitz, Allison Hoffman, Anthony Sacco, Norma Coe

**Affiliations:** 1 University of Pennsylvania, Philadelphia, Pennsylvania, United States; 2 University of Pennsylvania, University of Pennsylvania, United States; 3 University of Pennsylvania, University of Pennsylvania, Pennsylvania, United States

## Abstract

This study aims to understand the correlation between the legal framework around planning for medical treatment and the intensity of care received at the end of life. Advance directives (AD) allow an individual to legally document their wishes for medical treatment and end-of-life care, and durable powers of attorney (DPOA) allow them to identify an individual to make medical decisions on their behalf in the event they are no longer able. These laws vary greatly across time, place, and in their complexity. We estimated fixed effects model, which controls for time-invariant and state-specific factors affecting end-of-life care. Our primary outcome variable is the number of inpatient hospital days during the last six months of life, sourced from the Dartmouth Atlas Project. Our explanatory variables are hand-collected state-level legal statutes including whether the state (a) has default surrogate laws, (b) recognizes the ability to create a DPOA and/or AD, (c) permits oral directives, (d) authorizes a combined AD/DPOA, and (e) provides an official registry for ADs. Preliminary findings show recognizing DPOA and AD are negatively correlated with our outcome variable (-2.6 days; Std err 0.272) as are having default surrogate laws (-0.36 days; std err 0.157). However, allowing oral directives had an opposite effect (-.46 days; std err 0.157). Neither authorized combined AD/DPOAs nor AD registries resulted with significant correlations. These findings indicate that the existence of a basic framework such as default surrogate laws and a law governing ADs are key policy features correlated with reduced intensity in end-of-life care.

